# Montjuïc Hill (Barcelona): A Hotspot for Plant Invasions in a Mediterranean City

**DOI:** 10.3390/plants12142713

**Published:** 2023-07-21

**Authors:** Neus Ibáñez, Carlos Gómez-Bellver, Paula Farelo, Josep Maria Montserrat, Samuel Pyke, Neus Nualart, Jordi López-Pujol

**Affiliations:** 1 Botanic Institute of Barcelona (IBB), CSIC-Ajuntament de Barcelona, 08038 Barcelona, Catalonia, Spain; 2 Department of Evolutionary Biology, Ecology and Environmental Sciences, Faculty of Biology, University of Barcelona, 08028 Barcelona, Catalonia, Spain; 3 Barcelona Botanic Garden (JBB), Museum of Natural Sciences of Barcelona, 08038 Barcelona, Catalonia, Spain; 4 Escuela de Ciencias Ambientales, Universidad Espíritu Santo (UEES), Samborondón 091650, Ecuador

**Keywords:** alien flora, gardens, invasions, Mediterranean Basin, urban areas

## Abstract

Cities are often hotspots for biological invasions, showing much higher percentages of alien species than non-urbanized settings. The reasons are multiple and are mostly related to two main factors: their heterogeneous, highly disturbed habitats and their many gateways that allow alien species introduction (e.g., airports, roads, train stations, or gardens). In addition to being a sink of biological invasions, cities can also be a source of the spread of alien species into surrounding landscapes, which adds further complexity to this issue. Herein, we are presenting the results of a five-year survey of the alien flora of Montjuïc, the largest urban hill in Barcelona (Spain). In just about 3.4 km^2^, we recorded up to 247 alien plant taxa, a figure much higher than those of many other Mediterranean cities and which clearly points to the role of Montjuïc as a hotspot for alien plants. The comparison with the alien flora of its surrounding region (coastal Catalonia) suggests that the alien flora of Montjuïc would have become enriched through many immigration episodes from close geographic areas. The hill, however, would have also acted as a source of the spread of alien plants, and indeed, some species have not been detected yet beyond the confines of Montjuïc. This study aims to be a key tool to ensure early detection and also to develop appropriate management and/or eradication actions.

## 1. Introduction

It is widely recognized that urbanization is a major factor promoting biological invasions worldwide. Cities are hotspots for biological invasions, and many urban areas have a higher percentage of invasive species than other areas. Cities contain environmentally heterogeneous, highly disturbed habitats that potentially provide many ecological niches for alien species [1,2,3,4]. Urban areas often act as immigration gateways where alien species can enter—and later establish—through airports, harbors, and train stations, and also by means of markets, gardens (both public and private) and urban parks [5,6]. Horticulture is by far the main pathway for the introduction of alien plants into cities [7]. However, in addition to acting as a “sink”, cities can also be a “source” of the introduction of alien species into surrounding landscapes (e.g., [1,8,9,10,11]). In addition, several land use and socio-economic factors, such as disturbance or ruderalization, greenery, distance to city center, human population size, income, length of roads and railways, and GDP, have been shown to be associated with the success of non-native species in urban areas [12,13,14,15,16,17].

Given that the world’s urban population is rapidly expanding (the percentage of urban population has risen from 30% to 55% during the period 1950–2018, and it is expected that it might reach 68% by 2050; [18]), the problems derived from plant invasions associated with urban settings are expected to grow at the same pace. The urbanization of Catalonia (NE Spain) is not an exception to this trend; the population of the main metropolitan area of Catalonia, the Barcelona Metropolitan Region (BMR), increased from 863,000 in 1900 to 4,917,000 people in 2022 [19] thanks to an enormous increase in the built-up area (from <20 km^2^ in 1880 to >600 km^2^ at present; [20]). 

**Figure 1:**
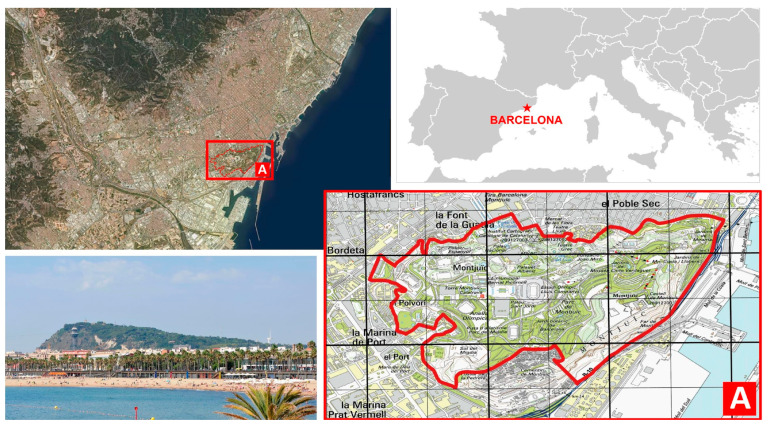
Location of Montjuïc hill (Catalonia, Barcelona, NW Spain). Upper right, location of Barcelona (credit: Wikimedia Commons). Upper left, aerial picture of Barcelona (credit: Institut Cartogràfic i Geològic de Catalunya, under CC BY 4.0), with the red box containing the study area. Lower right, red line delimiting the study area (i.e., the area covered by the city masterplan for Montjuïc hill, about 3.38 km^2^) (credit: Institut Cartogràfic i Geològic de Catalunya, under CC BY 4.0). The grid cells are of 500 × 500 m. Lower left, photo of Montjuïc hill from the NE (credit: Ralf Roletschek, under GFDL-1.2-only; https://commons.wikimedia.org/wiki/File:14-08-05-barcelona-RalfR-041.jpg; accessed on 15 February 2023).

Within the BMR, we have selected Montjuïc hill as a case study to check the role of urban areas as “hotspots” for plant invasions, given its dual role as both sink and source (see above). This, Barcelona’s most emblematic hill, is located at the south-west tip of the city (but at the core of the BMR), has a total area of about 3.4 km^2^ and a maximum altitude of 173 m (Figure 1). Nowadays, Montjuïc can be defined as an “urban hill”, as a large part of it is urbanized (43%), with a plethora of sport facilities, schools, museums, and exposition centers, and also with some residential areas. About 33.4% are managed green spaces (urban parks, botanical gardens and nurseries), while the remaining 23.6% are natural or semi-natural areas, including abandoned fields, forest patches (mainly of *Pinus* spp.), dry meadows, cliffs and slopes (with some remnants of *Quercus coccifera* L. formations), and ancient quarries that have been colonized by spontaneous vegetation [21]. We believe, thus, that Montjuïc is an ideal case study of the role of urban areas as a sink/source of alien species for a series of reasons that can be summarized as follows: (1) deep landscape modification; (2) occurrence of multiple gateways for alien species; and (3) presence of mass tourism.

Montjuïc hill can be taken as an example of how biological invasions are quickly progressing in urban or semi-urban environments, and hence, it could be regarded as a natural laboratory for invasions, particularly in a Mediterranean context, as all the above-mentioned factors can facilitate the introduction and the establishment of alien species. The Mediterranean Basin is one of the world’s hotspots for plant diversity [22]. A very large part of the Mediterranean flora are narrow endemics and, therefore, very threatened species [23]. In addition, the basin is also home to multiple phylogeographic refugia that harbor unique, distinct genetic lineages [24], so the risk posed by alien plants is potentially catastrophic. The main objective of this paper is to obtain a comprehensive catalog of the alien flora of this urban hill, which will allow us to characterize their life-history traits, geographical origin, taxonomic circumscription, invasive stage, and introduction pathway, among others. Furthermore, the alien flora of Montjuïc is compared with the alien flora of its surrounding region (Catalonia; [25]) regarding these characteristics. Compiling a list of the alien species of a given territory (as a checklist, or better, as a comprehensive catalog) is the first step in identifying the patterns of biological invasions and a key tool within an early detection strategy, and it may also help to develop appropriate management and/or eradication strategies [14,26,27].

## 2. Material and Methods

### 2.1. Study Area

Montjuïc has a complex orography due its being an irregular cone actually formed by three hillsides, as can be observed in Figure 1. Although the SW part of Montjuïc ends in an impressive cliff of ca. 170 m facing the sea (*Morrot*) that was produced by a fault, there are many cliffs (including quarry “scars”) scattered across the hill; indeed, Montjuïc soon became the main quarry of the region (since the Iberian and Roman periods and up to the middle 20th century; [21]). Montjuïc hill is composed of sedimentary rocks (conglomerates, sandstones, mudstones, and marlstones) deposited in a delta during the Middle Miocene [28]. The situation of Montjuïc defines its Mediterranean climate, which is characterized by dry and hot summers as well as wet and mild winters; the precipitations are limited and irregular, and they are concentrated in spring and autumn (the average annual temperature on Montjuïc is ca. 16.5 °C, while the annual precipitation is about 620 mm; https://www.worldclim.org/; accessed on 31 January 2022). 

The study area is delimited by the city masterplan for Montjuïc hill, which covers 3.38 km^2^ (Figure 1; see also [21]). The UTM (Universal Transverse Mercator) coordinate system has been used for geolocating the plants included in the study.

### 2.2. Data Gathering, Plant Identification, and Analyses

In order to produce a catalog of the alien flora of Montjuïc hill, we have combined fieldwork and a literature search. Regarding the fieldwork, we have prospected the whole area by conducting countless field trips from 2016 to 2020. Areas with evident management, such as urban parks and botanic gardens, have not been prospected. Plants from abandoned parks/gardens (i.e., persisting from cultivation) have not been included unleaccss they show sexual or asexual reproduction and clear signs of escape (i.e., they can be regarded as casual plants). Vouchers for some species (particularly new alien species at the regional/local levels) have been deposited in the BC herbarium in order to ease their correct identification and to have a testimony for future checking. Regarding the literature review, we have focused on four main sources: (i) the checklists arising from the 2015–2018 editions of the Barcelona BioblitzBCN (still unpublished or only partially published), which took place in Montjuïc; (ii) unpublished records of researchers from our team, mostly from the period 2000–2015; (iii) published studies that are focused on or include this area (including regular papers in journals, monographs, local and regional catalogs, and floras); and (iv) databases covering the studied area, especially the BDBC (Biodiversity Data Bank of Catalonia) [29] and the GBIF [30]. In addition, herbarium material was revised in the BC and BCN herbaria (acronyms according to Index Herbariorum [31]).

We identified plants based on our experience, by consulting relevant floras (i.e., floras that cover the regions that provide most of the alien plants in our area; e.g., in the web portal eFloras.org (http://www.efloras.org/; accessed on 16 May 2016) there are several online floras of the Americas as well as *Flora of China*) and monographs focused on a given taxonomic group and other specialized works (e.g., *Agaves of Continental North America* [32] or Manual of the Alien Plants of Belgium [33]), and also by checking with experts in certain taxa or territories (e.g., Gideon Smith from Nelson Mandela University for Crassulaceae or Léia Scheinvar from Universidad Nacional Autónoma de México for *Opuntia*). 

As a general rule, we have regarded as “alien” all those plant taxa occurring in Montjuïc that are listed in the recent checklist of the vascular alien flora of Catalonia [25]. For those taxa not included in this checklist, we considered as “alien” those that are not listed as autochthonous in Catalonia according to Bolòs and Vigo [34], Bolòs et al. [35] and Sáez and Aymerich [36]. The names of taxa included in the Montjuïc catalog have been updated according to the Tropicos (https://tropicos.org/name/Search; accessed on 1 January 2021) and Plants of the World Online (https://powo.science.kew.org/; accessed on 1 January 2021) databases as well as the checklist of Aymerich and Sáez [25]. In addition, we revised the most recent advances in plant phylogeny and taxonomy released in the last few years for some specific controversial taxa. The families of each taxon have been assigned according to APG IV [37].

To gain insights into the alien flora of Montjuïc hill, we have compared our catalog with the alien taxa of Catalonia that are considered to occur at present in coastal areas—885 taxa in total (see Aymerich and Sáez [25]). To do so, for each alien species occurring on Montjuïc, we have assigned the category to which it belongs by a series of traits following the scheme proposed by Aymerich and Sáez [25] (see this reference for a detailed description of all the categories):

1. native range (eleven categories): Mediterranean, Western Palearctic, Eastern and Southern Asia, Tropical Africa, South Africa, Australasia, (sub)tropical regions, North America, South America, cultivated (including artificial hybrids), and spontaneous hybrids;
2. plant growth type (ten categories): annual grasses, annual forbs, perennial grasses, perennial forbs, bulbous monocots, aquatic plants, climbers, succulent plants, shrubs and trees;
3. degree of naturalization in Catalonia (three categories): casual, naturalized and invasive;
4. relative abundance in Catalonia (four categories): rare, scattered, locally abundant and common;
5. residence time in Catalonia (four categories): before 1500 AD, 1500–1900, 1900–1970, or after 1970;
6. introduction pathway in Catalonia (four categories): agriculture, gardening, forestry and trade.

The life history and all the data for the alien taxa of Montjuïc not included in Aymerich and Sáez [25]—either because they are new to Catalonia or because the taxonomic criteria are not the same—have been compiled from monographs and floras as well as from databases, including the GRIN (https://www.ars-grin.gov/; accessed on 1 January 2021), Global Invasive Species Database (GISD; http://www.iucngisd.org/gisd/; accessed on 1 January 2021), Invasive Species Compendium (CABI; https://www.cabidigitallibrary.org/product/qi; accessed on 1 January 2021), and Pacific Island Ecosystems at Risk (PIER; http://www.hear.org/pier/; accessed on 1 January 2021). 

To obtain an updated, reliable catalog of the alien flora of Montjuïc hill, we have excluded all the plants not observed after 2000. This date represents approximately the hill’s last important transformation. 

## 3. Results

### 3.1. Alien Plants in Montjuïc: Amount

**Figure 2:**
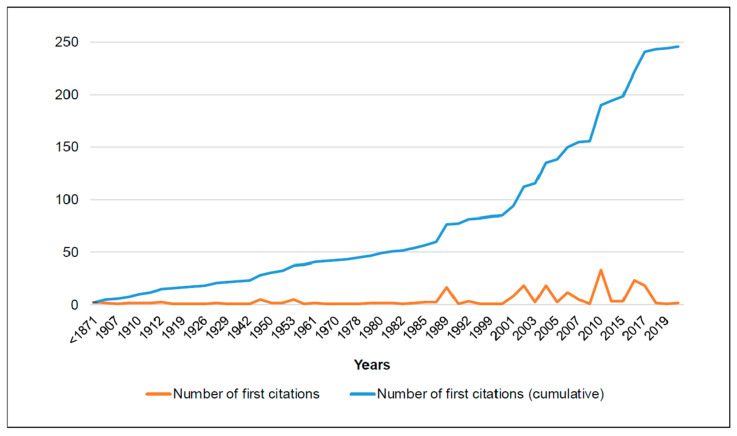
Distribution of the number of first citations of the alien plant taxa observed in Montjuïc, both non-cumulative (orange line) and cumulative (blue line).

The alien flora identified for Montjuïc included a total of 247 taxa (Supplementary Table S1; for the taxonomic considerations, see Supplementary Text S1), which represent ca. 27.9% of the alien flora of coastal Catalonia (885 taxa; [25]). Not included among these 247 taxa are (i) up to 21 taxa that have been cited prior to the year 2000 without further observations in Montjuïc (Supplementary Text S2); and (ii) five taxa that are considered as persisting plants from cultivation (Supplementary Text S2). Although most of the 247 taxa included in our catalog are included in the list of alien coastal areas of Catalonia by Aymerich and Sáez [25], there are a further seven neither published in the latter checklist nor published in any manuscript or database so far (Supplementary Text S3). Regarding the year of first citation, nearly 60% of the new observations of alien plants in Montjuïc have been made after the year 2000, and during the last four years (2017–2020), up to 24 new taxa have been found (Figure 2).

### 3.2. Alien Plants in Montjuïc: Patterns

**Figure 3:**
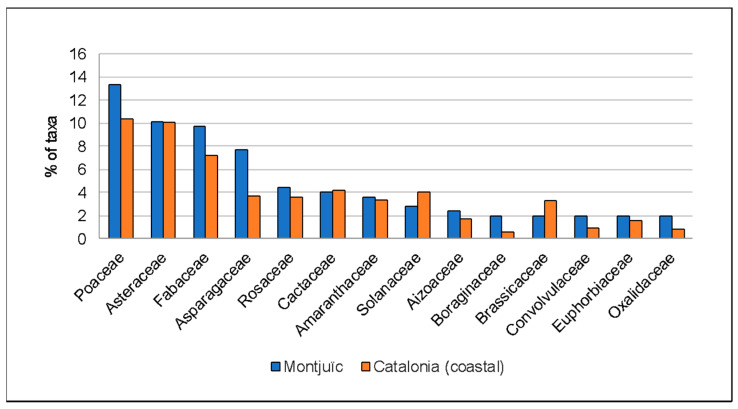
Distribution of the number of first citations of the alien plant taxa observed in Montjuïc, both non-cumulative (orange bar) and cumulative (blue bar).

The alien flora of Montjuïc includes taxa of 63 families (out of the 115 families that are found in coastal Catalonia, according to Aymerich and Sáez [25], i.e., 54.8%), only three of them with more than 20 taxa (Poaceae, Asteraceae and Fabaceae). These three families are also the most diverse ones if we take into account the whole alien flora of the Catalonian coastal areas, with similar percentages (Figure 3). Asparagaceae, Rosaceae, Cactaceae, Amaranthaceae and Solanaceae are the following families for both Montjuïc and coastal Catalonia, with the only difference being that Asparagaceae is rather more frequent in Montjuïc (7.7% in Montjuïc vs. 3.8% in coastal Catalonia; Figure 3). The alien flora of Montjuïc has representatives of up to 176 genera (473 for coastal Catalonia, according to Aymerich and Sáez [25]), with *Opuntia*, *Agave* and *Oxalis* being those with more than five taxa (for coastal Catalonia, these three genera rank first, third and nineteenth; see Supplementary Table S1). 

**Figure 4:**
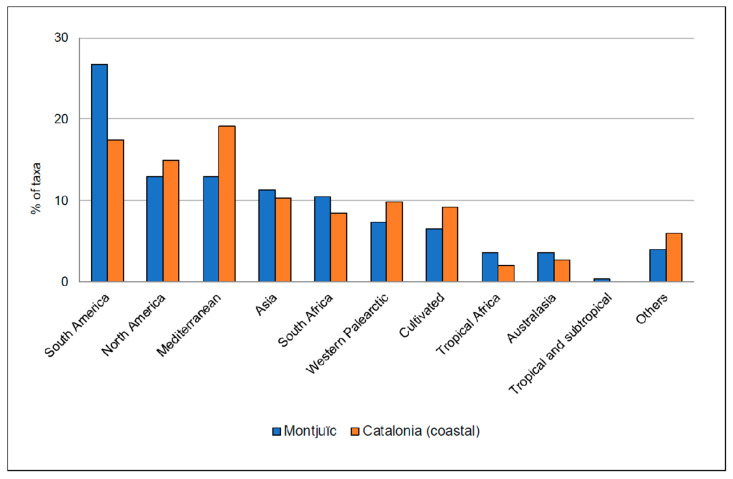
Geographic origin (native range) of the alien plant taxa observed in Montjuïc (present work, in blue) and in coastal Catalonia ([25]; in orange). The taxa are classified into one of the following categories: Mediterranean, Western Palearctic, Eastern and Southern Asia, Tropical Africa, South Africa, Australasia, tropical and subtropical regions, North America, South America, cultivated (including artificial hybrids), and spontaneous hybrids.

Regarding the geographic origin (Figure 4), the alien flora of Montjuïc is diverse and includes all the native origin categories except the spontaneous hybrid origin. Nearly 40% of all the taxa are from America (26.7% from South America and 13.0% from North America). Compared with the geographic origin of the taxa present in Catalonian coastal areas according to Aymerich and Sáez [25], the percentage of taxa from America is higher in Montjuïc (39.7% vs. 32.6%), although that of Mediterranean taxa is lower (13.0% vs. 19.1%; this origin ranks first for coastal Catalonia). The percentages of the other main origins are similar: 11.3% vs. 10.3% from Asia, 10.5% vs. 8.5% from South Africa, 7.3% vs. 9.8% from Western Palearctic, and 6.5% vs. 9.3% are plants of cultivated origin (Figure 4).

**Figure 5:**
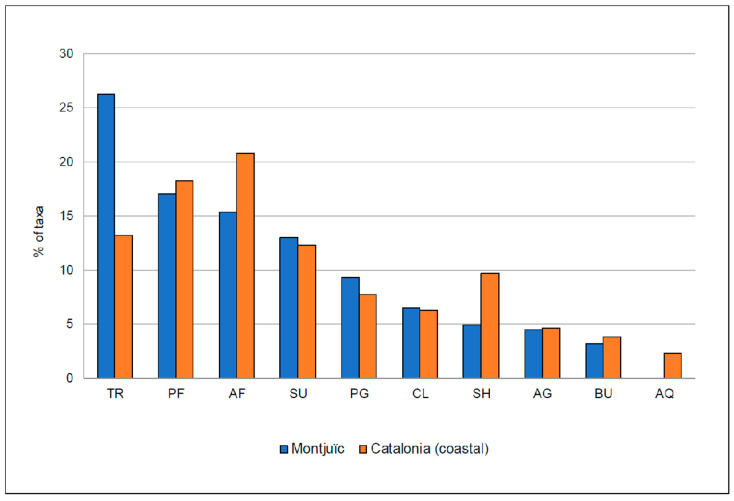
Plant growth types of the alien plant taxa observed in Montjuïc (present work, in blue) and in coastal Catalonia ([25]; in orange). The taxa are classified into one of the following categories: AG, annual grasses; AF, annual forbs; PG, perennial grasses; PF, perennial forbs; BU, bulbous monocots; AQ, aquatic plants; CL, climbers; SU, succulent plants; SH, shrubs; and TR, trees.

The most frequent plant growth type (Figure 5) observed on Montjuïc is trees (26.3%), followed by perennial forbs (16.6%), annual forbs (15.4%), and succulents (13.0%); bulbous monocots are less frequent (3.2%). For the coastal areas of Catalonia, in contrast, the most frequent types are annual forbs (20.8%), perennial forbs (18.3%) and trees (13.2%). 

**Figure 6:**
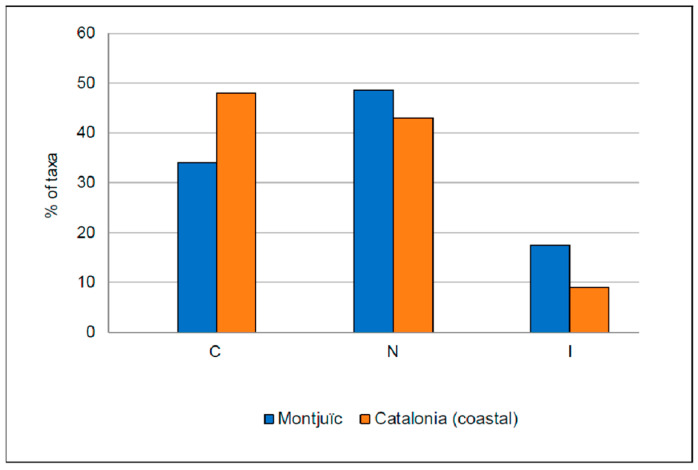
Degree of naturalization of the alien plant taxa (percentage) observed in Montjuïc (in blue) and in coastal Catalonia (in orange). The taxa are classified into one of the following categories: C, casual; N, naturalized; and I, invasive. Data on the degree of naturalization for both Montjuïc and coastal Catalonia are for the whole Catalonia level, and they have been taken from Aymerich and Sáez [25].

According to the degree of naturalization of the alien plants (data extracted from Aymerich and Sáez [25]), about half of the known taxa in Montjuïc (48.6%, 120 taxa) are naturalized aliens, 84 are casual (34.0%) and 43 are invasive (17.4%) (Figure 6). For the coastal areas of Catalonia, in contrast, casual plants are prevalent (48.0% vs. 34.0% for Montjuïc), followed by naturalized (42.9% vs. 48.6%) and invasive (9.0% vs. 17.4%) (Figure 6).

**Figure 7:**
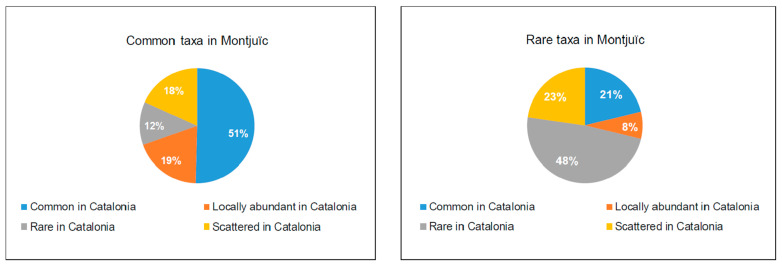
Relative abundance in Catalonia of the common (**left**) and rare (**right**) alien plant taxa in Montjuïc. The rare taxa in Montjuïc are those with only a few detected populations, while the common taxa have many populations on the hill (own work). The assignment to the four categories for the relative abundance in Catalonia (rare, scattered, locally abundant and common) has been taken from Aymerich and Sáez [25].

According to the relative frequency of the taxa in Montjuïc, about half of them are rare (53.4%) while the other half are regarded as common within the hill (46.6%). Of the common taxa in Montjuïc, 50.4% are considered common in Catalonia, 19.1% locally abundant, 12.2% rare, and 18.3% scattered (Figure 7). Of the taxa that are rare in Montjuïc, 21.2% are considered common in Catalonia, 7.6% locally abundant, 48.5% rare, and 22.7% scattered (Figure 7). In Supplementary Text S4, we have included the common taxa in Montjuïc that are rare in Catalonia, and in Supplementary Text S5, the rare taxa in Montjuïc that are common in Catalonia.

**Figure 8:**
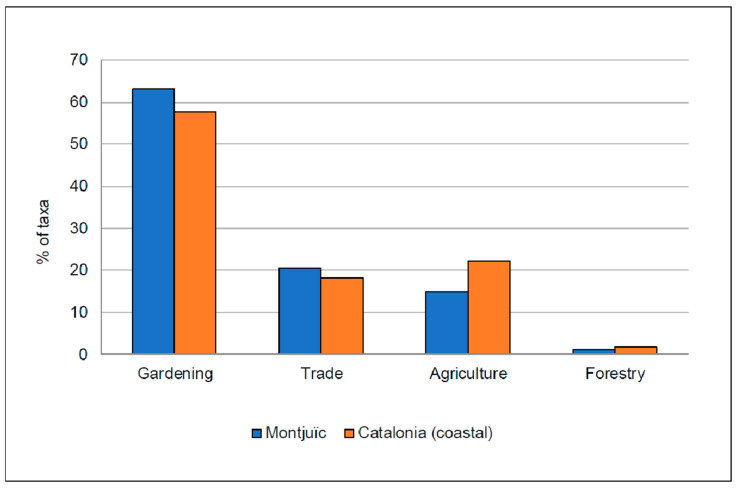
Introduction pathways of the alien plant taxa observed in Montjuïc (in blue) and in coastal Catalonia (in orange). The taxa are classified into one of the following categories according to Aymerich and Sáez [25]: agriculture, gardening, forestry and trade.

In relation to the way of introduction (data extracted from Aymerich and Sáez [25]), most of the taxa in Montjuïc were introduced in Catalonia for gardening purposes (63.2%, 156 taxa), followed by trade (20.6%, 51 taxa), agriculture (15.0%, 37 taxa), and forestry (1.2%, only three taxa) (Figure 8).

## 4. Discussion

Montjuïc, the largest urban hill in Barcelona, has a very rich alien flora, as could be expected for an urban area [10,15,38]. In <3.4 km^2^, we have been able to detect up to 247 alien plant taxa. This figure is even more astonishing if we compare it with those of other (always much larger) urban areas, both within and close to the Mediterranean Basin. For example, Montjuïc is much richer in alien plants than Mostar (152 taxa in 20 km^2^; [39]) and Sarajevo (82 taxa in 32 km^2^; [40]) in Bosnia, Podgorica in Montenegro (172 taxa in 86 km^2^; [41]), Thessaloniki in Greece (147 taxa in 61 km^2^; [42]), and Palermo (170 taxa in 63.5 km^2^; [43]) and Rome (228 taxa in 1287 km^2^; [44]) in Italy. Therefore, the role of Barcelona’s “magic hill” (as it is often termed by local residents) as a hotspot for alien plants is indisputable, and it may stem from being both a sink and a source of alien plants. 

Although it will be discussed in more depth later, the alien flora of Montjuïc hill has probably been enriched through the arrivals of multiple species from neighboring areas. The coastal strip of Catalonia, with an area of 14,000 km^2^, harbors 885 taxa, while the figure rises to 1068 for the whole of Catalonia (ca. 32,000 km^2^) [25]. Coastal Catalonia—and the whole of Catalonia—is a hotspot for alien plants itself, as it has a much larger flora than other regions of a similar size (or even of a much greater size), encompassing some of the cities mentioned above: Lazio region (which includes Rome) has 526 alien plant taxa in ca. 17,000 km^2^ [45], while Greece (which includes Thessaloniki) has just 343 alien taxa in 132,000 km^2^ [46]. The reasons may include (i) a mild climate that allows the establishment of temperate but also subtropical and tropical species; (ii) the constant alteration of its landscape, mostly thanks to having experienced important industrialization since the 19th century and a high urbanization rate, especially in its coastal areas, which peaked with the “Spanish real estate bubble” at the turn of the 21st century [47]; and (iii) the great diversity of habitats within a relatively small territory, which makes possible the introduction of many plants showing very different functional traits.

Evidence supporting the role of Montjuïc as a sink of plant invasions from coastal areas of Catalonia comes from comparing their alien flora. While the vast majority of the 247 plant taxa has also been recorded in coastal Catalonia, the two alien flora have a similar taxonomic profile (they share most represented families and genera) and geographic origin (America as the largest source of taxa), in addition to close patterns of relative abundance (common taxa in Montjuïc are also common in coastal Catalonia, and the same can be applied for rare taxa) and of introduction pathways (gardening as the main way of introduction). The flora of Montjuïc, nevertheless, cannot be regarded as a sample of the flora of coastal Catalonia due to some important differences that need to be noted. First, the slightly higher percentage of plants with ornamental uses in Montjuïc (63.2% vs. 58.0%) is likely due to the large number of (mostly) public gardens present on the hill (about one-third of the area of Montjuïc (33.4%) is managed green spaces); thus, many alien plants may occur in Montjuïc as garden escapes. The many gardens of Montjuïc hill can also be linked to its higher percentage of American plants compared to coastal Catalonia (39.7% vs. 32.6%); indeed, a large fraction of the ornamental flora of the northwestern Mediterranean Basin is of American origin. Guillot [48] provided a list of the ca. 200 most cultivated ornamental plants in Spain, and among the alien taxa, 40.9% were of American origin. Finally, the much higher percentage of trees in Montjuïc compared to coastal Catalonia (26.3% vs. 13.2%) seems, again, to be associated with Montjuïc’s gardens. Taking again the compendium of Guillot [48], up to 36.0% of the most common ornamental alien species are trees.

**Figure 9:**
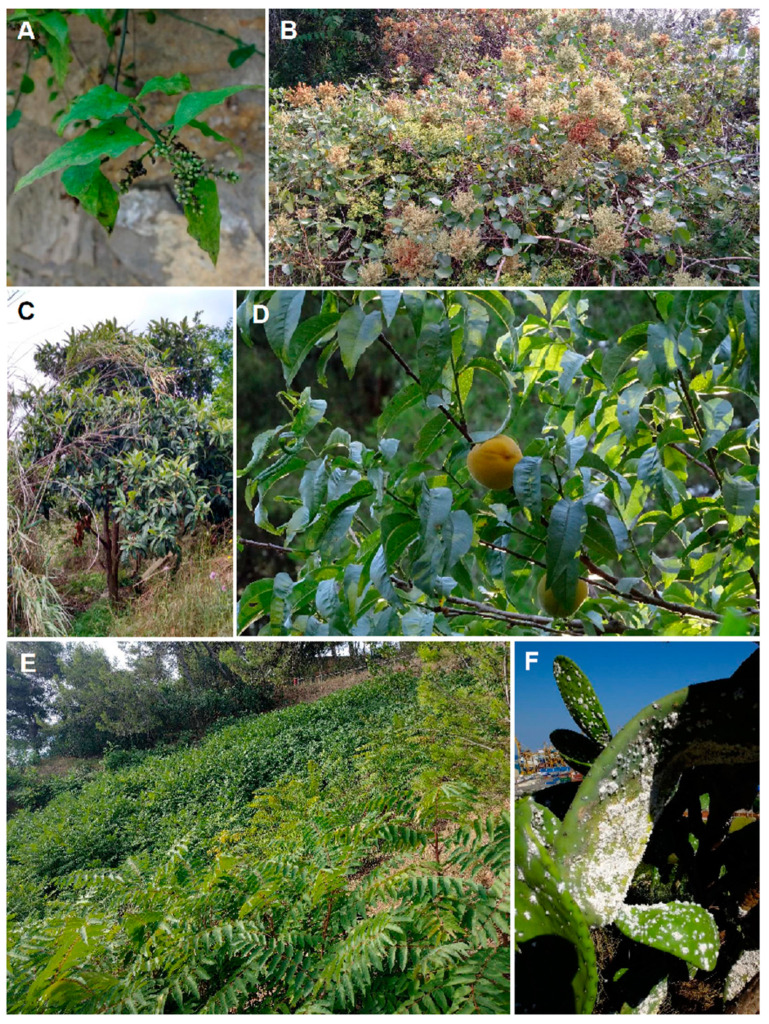
Images of some examples of the alien taxa detected in Montjuïc hill. (**A**) *Bosea amherstiana* (20 June 2016); (**B**) *Rumex lunaria* (18 May 2016); (**C**) *Eriobotrya japonica* (18 May 2016); (**D**) *Prunus persica* (21 August 2019); (**E**) *Ailanthus altissima* (28 September 2022); and (**F**) *Opuntia ficus-indica* attacked by *Dactylopius opuntiae* (16 June 2017). All the photographs were taken by Jordi López-Pujol.

In addition to being a sink, Montjuïc could act (and possibly did in the past) as a source of alien plants for the neighboring areas. There are up to seven plant taxa neither included in the list of alien coastal areas of Catalonia [25] nor published in any manuscript or database so far. Two of them represent new records for continental Europe (*Bosea amherstiana* Hook. f. (Figure 9A) and *Digitaria radicosa* (J. Presl) Miq.), one for the Iberian Peninsula (*Rumex lunaria* L.; Figure 9B), and four for Catalonia (*Echium candicans* L. f., *Furcraea selloana* K. Koch, *Pandorea jasminoides* (Lindl.) K. Schum., and *Ruscus hypophyllum* L.). In addition, there are eight taxa that, although listed by Aymerich and Sáez [25], were firstly observed in Montjuïc in recent years (since 2011–2018): *Aristolochia sempervirens* L., *Enneapogon cenchroides* (Licht. ex Roem. and Schult.) C. E. Hubb., *Morus kagayamae* Koidz., *Opuntia elatior* Mill., *Paraserianthes lophantha* (Willd.) I. C. Nielsen, *Ptelea trifoliata* L., *Tara spinosa* (Feuillée ex Molina) Britton and Rose, and *Vachellia caven* (Molina) Seigler & Ebinger (Supplementary Text S3). Indeed, six out of the eight taxa were observed thanks to our fieldwork on the hill since 2016. Most of these “new” species are cultivated in gardens in Montjuïc, including the Botanical Garden of Barcelona (opened in 1999), the old Acclimatization Gardens of 1930, and the Gardens of Mossèn Costa i Llobera (the latter, built in 1970, specializes in cacti and other succulent plants). For example, *Bosea amherstiana*, which is native to north Pakistan through to the central Himalaya, has long been cultivated in several Mediterranean acclimatization/historic gardens in addition to those of Barcelona (e.g., [49,50]). Botanical gardens, in spite of playing very important social and scientific roles, are well-known sources of plant invasions [51]. According to Hulme [52], botanical gardens would be the most probable source of the introduction to over half of the world’s worst invasive species. About half (124 taxa) (Supplementary Table S1) of the alien taxa detected in our study are cultivated in Montjuïc’s gardens. Therefore, although of course proof of causality cannot be proven, Montjuïc’s local gardens should be considered as a source of the wild alien flora of the hill, in addition to immigration from coastal areas of Catalonia. Albeit not a garden, Montjuïc Cemetery, the largest in the city (of over 0.5 km^2^), is located on the SW part of the hill. The cemetery’s flora may contain high proportions of alien species [53] that could escape.

The role of Montjuïc as a sink/source of alien plants is likely enhanced by other factors that are well-known drivers of alien plant establishment and also of expansion, namely land-use changes (i.e., intense anthropogenic disturbances) and the movement of goods and people [54,55]. Regarding the first factor, we should note that the hill has been severely modified since prehistoric times. Although human presence has been recorded since the Paleolithic [56], the largest transformation has occurred since the late 18th century. At the top of the hill, a large military fortress (with over 10 ha) was built between 1751 and 1799, while in 1883, a large part of the hill was transformed into the above-mentioned cemetery. Meanwhile, quarry activities peaked during the late 19th–early 20th century (with over 100 ha; [57]). In addition, according to old documents (including old maps), large parts of the hill were cultivated extensively until the early 20th century, when the Universal Exposition of 1929 drove the modification of the lower part of the hill, which was urbanized to build the exposition facilities or transformed into urban gardens [21]. Some old records of alien plants in Montjuïc could be testimony to these human activities, with two taxa being worth mentioning that have been cited in Montjuïc before the date of introduction into Catalonia indicated by Aymerich and Sáez [25]: *Aloe maculata* All., which was cited as very abundant on the hill by Sennen in 1917 (as *Aloe umbellata* DC.) in his *Flore of Catalogne* [Flora of Catalonia] [58], and *Xanthium orientale* L., for which there is a herbarium specimen of 1871 (BC-612262). *Aloe maculata*, however, could have been present on the hill much before, as Colmeiro [59] indicates “cultivated and as spontaneous near the port [of Barcelona]”. In addition, although it appeared in Montjuïc probably after its introduction into Catalonia, *Agave americana* L. could have occurred as an escape as early as the first half of the 19th century, as it is often represented in engravings from that time [60,61,62], and the “hiking guide” to Montjuïc of 1899 also suggests that it was very common at that time [63]. For these three taxa (and possibly for other ones), Montjuïc would have acted as a suitable habitat for their naturalization and as a source of their spread. The several wild fruit trees (e.g., *Eriobotrya japonica* (Thunb.) Lindl. (Figure 9C), *Malus domestica* (Suckow) Borkh., or *Prunus persica* (L.) Batsch (Figure 9D)) we have observed on the hill could be remnants of the old orchards observed by Solé and Calvo [63], or alternatively, of those that accompanied the shanty towns, which occupied large parts of the hill in the 1950s (with up to 52,000 people in 1957 [57]) because of the massive immigration wave, mainly from southern Spain (it is estimated that about 1.4 million people migrated to Catalonia during the period 1950–1957 [64]). 

Although additional areas of Montjuïc hill were transformed into urban gardens during the 1960s and 1970s [21], the largest changes in the hill’s landscape were those of the 1980s and 1990s. Most venues for the 1992 Olympic Games were placed on the hill (the *Anella Olímpica*), and in recent years, additional urban parks and the city’s new botanical garden (Barcelona Botanical Garden, of ca. 14 ha) have been built within Montjuïc. The fact that nearly 60% of the new observations of alien plants in Montjuïc have been made after the year 2000 strongly suggests that the current alien flora is mostly the result of the recent transformation of the hill. In contrast, there are up to 21 alien taxa that have not been observed since the end of the last century. Some of these are species often associated with crops (which no longer exist on Montjuïc), such as *Abutilon theophrasti* Medik., *Glebionis segetum* (L.) Fourr., or *Hypericum triquetrifolium* Turra. In addition, some taxa with medicinal or alimentary uses (e.g., *Anagyris foetida* L., *Ervilia sativa* Link, *Trigonella foenum-graecum* L., or *Tropaeolum majus* L.) probably disappeared from the hill with the abandonment of the subsistence orchards associated with the demolition of the shanty towns in the 1960–1980s; for example, we were able to locate an old picture (from ca. 1925–1935) that shows *Tropaeolum majus* growing on the roof of a shack [65]. Other plants likely associated with the agricultural exploitation of Montjuïc, however, are still present, such as *Allium sativum* L. and *Triticum aestivum* L. (which are rare in the hill) or *Beta vulgaris* L. and *Vitis vinifera* L. (common in Montjuïc).

The close proximity to some of Barcelona’s main transport facilities and the high number of visitors represent additional factors enhancing the role of Montjuïc as a sink and source of plant invasions. The distance to airports and seaports, the occurrence of roads and railways, and the human population density are well-known drivers of species invasions [54,66,67]. The number of visitors, however, has also been shown to be related to the spread and establishment of alien species [68,69,70], as touristic and recreational activities usually involve the frequent congregation of people and vehicles from geographically diverse areas. The seaport of Barcelona is bordering Montjuïc along almost 3 km, while the ring-road expressway (*Ronda del Litoral*) and the railway to the seaport are also delimiting Montjuïc on its south-eastern side. In addition, Barcelona’s international airport (the seventh busiest airport in Europe; [71]) is just 6 km away. The city of Barcelona is one of the most visited cities in the world (ranking 17th) and the fifth most visited city in Europe (over 9 million international visitors yearly; [72]). The year just before the outbreak of COVID-19, there were about 34 million overnight stays by tourists [73]. Within Barcelona, Montjuïc has become one of the main tourist attractions. In 2008, it was estimated that 17 million people visited Montjuïc [21], so the present figures are probably much higher. Some of the city’s most visited sites are located on the hill, such as Poble Espanyol de Barcelona (1.24 million visitors in 2019), Montjuïc Castle (0.88 million), National Art Museum of Catalonia (0.84 million), CaixaForum Barcelona (0.67 million), and Joan Miró Foundation (0.36 million) [73]. The Montjuïc cable car, for example, had 1.62 million users in 2019 [73]. In addition, the main music venues and shows taking place within Montjuïc facilities (Olympic Stadium and Palau Sant Jordi Arena) had a total of 0.65 million spectators in 2018 [74]. The “Vision 2029”, which aims to attract the local people of Barcelona to the hill at the time of the 100-year jubilee of the Universal Exposition of 1929 [75], will likely increase the number of visitors.

## 5. Conclusions and Conservation Implications

As we anticipated, Montjuïc hill in Barcelona is a hotspot for alien plants, as in a very small area (<3.4 km^2^), there are 247 alien plant taxa, which represents almost one quarter of the total alien flora of the whole of Catalonia (with ca. 32,000 km^2^). The body of alien flora of Montjuïc is, surprisingly, considerably larger than those of many other Mediterranean cities (e.g., Rome). The extremely rich alien flora of Montjuïc is the result, on the one hand, of the immigration of species from surrounding areas (coastal Catalonia is itself a hotspot for alien plants), and on the other hand, of the establishment of species that have escaped from the many gardens located on the hill. The highly human transformation of Montjuïc during the last two centuries (e.g., the construction of the venues for the Universal Exposition of 1929 and the Olympic Games of 1992, and the sprawl of shanty towns in the 1950s) would have helped much with this process. 

Managing the alien species in Montjuïc is a complex task because of the many uses of the hill (as a tourist attraction, as a place for leisure and other recreational activities, e.g., cycling, hiking, or as a cultural destination). Although new urban development is not planned for the hill, some economic and cultural lobbies are piling on pressure to increase the number of visitors to Montjuïc [76,77]. In addition, Futbol Club Barcelona will move to the Montjuïc Olympic Stadium during the season 2023–2024 (because of the renovation works to its own stadium; [78]), which will mean huge movements of people several times a month. Although the city council is planning to improve the management and conservation of the natural and semi-natural areas [79], urgent eradication actions are needed, particularly focused on the most aggressive neophytes. *Ailanthus altissima* (Mill.) Swingle and *Senecio angulatus* L. f. are particularly worrisome, as the areas invaded by these species have increased the most during the years of our study, according to our personal observations (Figure 9E). In contrast, there are two neophytes that were very problematic just some years ago but are now declining because of pests (*Agave americana* and *Opuntia ficus-indica* (L.) Mill. are attacked by *Scyphophorus acupunctatus* and *Dactylopius opuntiae*, respectively; Figure 9F). Probably the only way to contain the increase in human activities and also to ensure the adequate management of alien species is by turning the hill into a protected area (PA). The hill has enough biodiversity values to be declared as PA. For example, up to 105 bird species have been observed just in the *Morrot* cliffs area of Montjuïc, including some species of the Spanish Catalog of Endangered Species (e.g., *Athene noctua*, *Falco peregrinus*, or *Falco tinnunculus*; [80]; of the latter, the Montjuïc colony is one of the largest in Europe [81]). Regarding the native flora, the hill harbors threatened and/or protected plant species, such as *Succowia balearica* (L.) Medik. or *Chamaerops humilis* L.; for the latter, Montjuïc represents the northernmost distribution limit of this species in Spain [82].

Actions for the early detection, control, containment and eradication of alien species in Montjuïc are important, not just to protect species of conservation interest that occur in the hill, such as the above-mentioned *Succowia balearica* or *Chamaerops humilis*. As demonstrated here, Montjuïc has been a source of the spread of new alien plants into the NW Mediterranean Basin (Catalonia); some species introduced through Montjuïc are well-known invasive species elsewhere, such as *Opuntia elatior* or *Paraserianthes lophantha*. Catalonia has a very rich native flora (3460 taxa), which represents about half (ca. 53%) and one-third (ca. 30%) of the flora of the Iberian Peninsula and the whole of Europe, respectively [36]; about 7% of the native flora is threatened with extinction, with some of these plants being narrow endemics with just one or a few populations [83]. Montjuïc and other regional foci of spread (e.g., the Costa Brava in NE Catalonia [84]) should merit, thus, further attention by managers and policymakers.

## Data Availability

The raw data supporting the reported results can be found in Supplementary Table S1.

## Supplementary Materials

The following supporting information can be downloaded at https://www.mdpi.com/article/10.3390/plants12142713/s1, Text S1: Taxonomic considerations regarding the alien plant taxa observed in Montjuïc, Text S2: Alien plant taxa not included in the catalog of 247 taxa of Montjuïc (i.e., Supplementary Table S1), Text S3: Novelties for the catalog of alien plant taxa of Montjuïc thanks to our fieldwork, Text S4: Common taxa in Montjuïc that are rare in Catalonia, Text S5: Rare taxa in Montjuïc that are common in Catalonia; Table S1: Alien flora identified in Montjuïc. For each plant taxa, we include (1) the family; (2) the origin (ME, Mediterranean; WP, Western Palearctic; AS, Eastern and Southern Asia; AF, Tropical Africa; CA, South Africa, AU, Australasia; TRO, tropical and subtropical regions; NA, North America; SA, South America; CUL, cultivated; spontaneous hybrids); (3) the plant growth type (AG, annual grasses; AF, annual forbs; PG, perennial grasses; PF, perennial forbs; BU, bulbous monocots; AQ, aquatic plants; CL, climbers; SU, succulent plants; SH, shrubs; TR, trees); (4) the degree of naturalization in Catalonia (C, casual; N, naturalized; I, invasive); (5) the abundance in Montjuïc (R, rare; C, common); (6) the abundance in Catalonia (R, rare; S, scattered; L, locally abundant; C, common); (7) the residence time in Catalonia (1, before 1500 AD; 2, 1500–1900; 3, 1900–1970; 4, after 1970); (8) the introduction pathway in Catalonia (A, agriculture; G, gardening; F, forestry; T, trade); (9) the intentionality of the introduction in Catalonia (A, accidental; D, deliberate); (10), the oldest citation in Montjuïc; (11) the voucher information (if vouchered in one of Barcelona’s herbaria, BC and BCN); and (12) whether it is cultivated in one of the gardens of Montjuïc hill. An asterisk denotes that the information has been taken from Aymerich and Sáez [25].
