# Supplementary material for: Montjuïc Hill (Barcelona): A Hotspot for Plant Invasions in a Mediterranean City
Source: Plants (Basel). 2023 Jul 21;12(14):2713. doi: 10.3390/plants12142713 (PMC10384852; doi:10.3390/plants12142713)
Supplement: Supplementary file 1 [file plants-12-02713-s001.zip › Text S1.pdf]

Text S1. Taxonomic considerations regarding the alien plant taxa observed in Montjuïc hill.

S1.1. TAXA WITH DIFFERENT CRITERIA FROM THOSE OF THE RECENT CATALONIAN CHECKLIST (AYMERICH & SÁEZ, 2019).

Mercurialis annua **L.**

Species included in the checklist, considered to be an archeophyte, Western Palearctic in distribution, present as a casual species throughout Catalonia, common and naturalised. We do not regard this species as allochthonous to Catalonia. It is a taxon that shows a certain degree of phenotypical variation, in which some authors have observed varieties (Bolòs & Vigo, 1996), subspecies (Bolòs *et al*., 2005) or even distinct species (Güemes, 1997). This is due to a complex history of divergence, migration and genetic flow. At present, the existence of a *Mercurialis annua* polyploid complex is under consideration (Ma *et al*., 2019). We believe it prudent to follow the route of previous authors in considering this plant to be subcosmopolitan, and not foreign, to our area, as in the case of the revisions of allochthonous flora of Catalonia and Aragón (Casasayas, 1989; Sanz *et al*., 2009), where it is not included.

Hedera algeriensis Hibberd

A plant not appearing in the checklist, though in a wider sense likely to have been included within *Hedera maroccana* McAll., as regarded by some authors. The former species is native to the south of the Iberian Peninsula and Morocco, and the latter to the Mediterranean area of Algeria and Tunisia (Green *et al*., 2011). The differences in some characters observed in Montjuïc plants allow us to distinguish the two taxa (Ackerfield & Wen, 2002; McAllister & Rutherford, 2011).

Nothoscordum* × *borbonicum Kunth

Scattered across Montjuïc. This is an invasive plant that causes considerable problems in gardens of many countries worldwide. Of the two species of *Nothoscordum* present in Montjuïc this is the most robust one, and its ability to multiply vegetatively is clearly superior.

Nothoscordum nudicaule (Lehm.) Guagl.

Locally present in the western half of Montjuïc, for example, above Jocs del '92 street. This taxon and the hybrid *N.* × *borbonicum* sometimes receive a synthetic treatment within *N. gracile* (Dryand. ex Aiton) Stearn, but *N. nudicaule* is distinguished by its triangular stamens, tepal colour, and the production of fewer bulbils (Pyke, 2019).

Spartium junceum **L.**

A plant not included in the checklist, which we consider to be allochthonous to Catalonia. In the Iberian Peninsula it is native further south, and only in the Baetic range (Talavera, 1999), maybe extending to Albacete and Murcia. The date of its introduction is not known with precision, though it was apparently introduced as an ornamental plant. However, it is worth noting its use as a shrub for stabilising road and track embankments (Torre *et al*., 1990; MOPT, 1992), in particular in the last thirty years, a practice that has led to the establishment, locally, of many present populations.

S1.2. OTHER TAXONOMIC CONSIDERATIONS

***Asparagus aethiopicus* L.**

Asparagus densiflorus (Casasayas, 1989) has been recorded from the city of Barcelona, and probably Montjuïc, this being a plant with much smaller cladodes. We also had identified plants observed on Montjuïc (BC 912410, S. Pyke, 28/6/2011) under that same name, and also from the relatively close Parc del Laberint of Barcelona (BioBlitzBCN database, C. Gómez-Bellver, 14/5/2014). On revising material from BC and BCN we have observed the dense form of *A. densiflorus* (probably the variety 'Myersii') cultivated on the first series of steps above Montjuïc's Font Màgica, though it should not be considered casual, or wild, in Catalonia (Aymerich & Sáez, 2019), and the records under that name must be attributed to *A. aethiopicus*.

Erigeron canadensis L. var. ***glabratus*** A. Gray

[≡ *Conyza canadensis* (L.) Cronq. var. *glabrata* (A. Gray) Cronq.]

The recent revision of the subgenus *Conyza* (Pyke, 2020) recognises two varieties within the territory: the type variety, of a more interior distribution, and var. *glabrata*, present in the lowlands, and of a more coastal distribution. Following the criterion of the majority of authors, and also the Catalonian checklist (Aymerich & Sáez, 2019), we choose to refer this variety to the genus *Erigeron*.

Phyla nodiflora (L.) Greene

Observed at least twice on Montjuïc (N. Montes & S. Pyke, 8/5/2015; T. Garnatje et al., 12/5/2015, *vidi vivam*). This taxon is not included in the recent checklist for Catalonia (Aymerich & Sáez, 2019), but rather, another congeneric species: *Phyla canescens* (Kunth) Greene. Some authors reckon that these two taxa, in a wider sense, constitute but one species, whereas others have proposed *Ph. nodiflora* as a subcosmopolitan species which, in our territory, ought to be regarded as non-allochthonous. According to Gross et al. (2017), we are dealing with two species separated on the basis of (1) some morphological characters, (2) the distribution of their native areas, (3) climatic preferences and (4) their reproductive biology. We maintain the classic criterion of two separate species (Bolòs & Vigo, 1996; Bolòs *et al*., 2005), these having been introduced into this part of the Mediterranean region, as do the revisions of introduced flora for the Valencian territories (Sanz *et al*., 2011) and for Italy (Galasso *et al*., 2018).

Opuntia ficus-indica (L.) Mill.

We consider that the Montjuïc plants formerly cited as *Opuntia maxima* Mill. and *O. ficus-barbarica* ought to be referred at present to *O. ficus-indica* (L.) Mill., at least until further studies have been carried out. In the first instance, we are dealing with a validly published plant which, however, lacks an indication of its true origin and only contains the references of cultivated plants. *Opuntia ficus-barbarica* should be understood as a synonym. This is a widely cultivated species, with variable morphological characters. The extensive Montjuïc populations constitute plants of a morphotype consisting of cladodes narrower than in the form employed as a fruit tree, along with flowers with orange-red tepals (Guillot et al., 2009).

Parthenocissus quinquefolia (L.) Planch

Plant absent from the Catalonian checklist (Aymerich & Sáez, 2019). It is very similar to *Parthenocissus inserta* (A. Kerner) Fritsch, and both species have been observed on Montjuïc as casuals. They can be distinguished, among other morphological details, by the form of the tip of the tendrils when in contact with the substrate: adhesive discs in the case of *P. quinquefolia*, and not, or hardly, inflated (in the form of a golf stick head), but never forming discs, as in the case of *P. inserta*. We have observed both *P. inserta* and the forms with inflated tendril tips, bearing in mind that this latter case might correspond to hybrid forms.

Xanthium orientale.

X. strumarium of old herbarium sheets is corrected to *X. orientale*.

REFERENCES

Ackerfield, J. & Wen, J. 2002. A morphometric analysis of *Hedera* L. (the Ivy genus, Araliaceae) and its taxonomic implications. *Adansonia* 24(2): 197-212.

Aymerich, P. & Sáez, L. 2019. Checklist of the vascular alien flora of Catalonia (northeastern Iberian Peninsula, Spain). *Mediterranean Botany* 40(2): 215-242.

Bolòs, O. de & Vigo, J. 1996. *Flora dels Països Catalans 3*. Barcino, Barcelona.

Bolòs, O. de; Vigo, J.; Masalles, R.M. & Ninot, J.M. 2005. *Flora manual dels Països Catalans*. 3a ed. Ed. Pòrtic. Barcelona. 1310 p.

Casasayas, T. 1989. *La flora al·lòctona de Catalunya. Catàleg raonat de les plantes vasculars exòtiques que creixen sense cultiu al NE de la Península Ibèrica*. Tesi doctoral. Universitat de Barcelona, Barcelona. 880 p.

Galasso, G.; Conti, F.; Peruzzi, L.; Ardenghi, N.M.G.; Banfi, E.; Celesti-Grapow, L. *et al*. 2018. An updated checklist of the vascular flora alien to Italy. *Plant Biosystems* 37 p.

Green, A.F.; Ramsey,T.S. & Ramsey, J. 2011. Phylogeny and biogeography of ivies (*Hedera* spp., Araliaceae), a polyploid complex of woody vines. *Syst. Bot.* 36(4): 1114-1127.

Gross, C.L.; Fatemi, M.; Julien, M; McPherson, H. & Van Klinken, R. 2017. The Phylogeny and Biogeography of Phyla nodiflora (Verbenaceae) Reveals Native and Invasive Lineages throughout the World. *Diversity* 9, 20: 1-23.

Güemes, J. 1997. *Mercurialis* L. *In:* Castroviejo, S.; Aedo, C.; Benedí, C.; Laínz, M.; Muñoz, F.; Nieto, G. & Paiva, J. (eds.). *Flora iberica* 8. Real Jardín Botánico (CSIC), Madrid.

Guillot, D.; Laguna, E. & Rosselló, J.A. 2009. Flora alóctona valenciana: Familia *Cactaceae*. *Monografías de la revista Bouteloua* 5. 148 p.

Ma, W. J., Santos del Blanco, L., & Pannell, J. R. 2019. A new biological species in the Mercurialis annua polyploid complex: functional divergence in inflorescence morphology and hybrid sterility. *Annals of botany* 124(1), 165-178.

McAllister, H.A. & Rutherford, A. 2011. *Hedera* L.: 227–232. A: Cullen, J., Knees S.G. & Cubey, H.S. (eds.) *The European Garden Flora. A manual for the identification of plants cultivated in Europe, both out-of-doors and under glass. Vol. IV. Angiospermae-Dicotyledons. Aquifoliaceae to Hydrophyllaceae*. Second edition. Cambridge University Press. Cambridge.

MOPT. Anònim. 1992. *Manual de plantaciones en el entorno de la carretera*. Ministerio de Obras Públicas y Transporte (MOPT). 77 p.

Pyke, S. 2019. Nothoscordum Kunth (Amaryllidaceae, formerly Liliaceae or Alliaceae) in the NE Iberian Peninsula: a confusing denizen of parks and gardens. *Bouteloua* 28: 12-18

Pyke, S. 2020. Conyza Less. (Asteraceae): una valoración crítica basada en las poblaciones de Cataluña, España. *Collect. Bot*. 39: e005.

Sanz, M.; González, F. & Serreta, A. 2009. La flora alóctona de Aragón (España). *Bot. Complutensis* 33: 69-88.

Sanz, M.; Guillot, D & Deltoro, V. 2011. La flora alóctona de la Comunidad Valenciana (España). *Bot. Complutensis* 35: 97-130.

Talavera, S. 1999. *Spartium* L. *In*: Aedo, C.; Castroviejo, S.; Romero, C.; Sáez, L.; Salgueiro, F.J. & Velayos, M. (eds.). *Flora iberica* 7(1). Real Jardín Botánico (CSIC), Madrid.

Torre, J.R. de la; Gil, P.; García, J.I.; González, J.R., & Gil, F. 1990. *Catalogo de especies vegetales a utilizar en plantaciones de carreteras*. Ministerio de Obras Públicas y Urbanismo (MOPU). 497 p.
