# Supplementary material for: Montjuïc Hill (Barcelona): A Hotspot for Plant Invasions in a Mediterranean City
Source: Plants (Basel). 2023 Jul 21;12(14):2713. doi: 10.3390/plants12142713 (PMC10384852; doi:10.3390/plants12142713)
Supplement: Supplementary file 1 [file plants-12-02713-s001.zip › Text S2.pdf]

Text S2. Alien plant taxa not included in catalogue of 247 taxa of Montjuïc hill (i.e., Supplementary Table S1).

S2.1. OLD RECORDS (PRIOR TO YEAR 2000)

1. *Abutilon theophrasti* Medik.
2. *Acer negundo* L.
3. *Amaranthus albus* L.
4. *Amaranthus hybridus* L.
5. *Amaranthus spinosus* L.
6. *Amaranthus viridis* L.
7. *Anagyris foetida* L.
8. *Ballota hirsuta* Benth.
9. *Buddleja davidii* Franch.
10. *Cyrtomium falcatum* (L. f.) C. Presl
11. *Elodea canadensis* Michx.
12. *Ervilia sativa* Link
13. *Glebionis segetum* (L.) Fourr
14. *Hypericum triquetrifolium* Turra
15. *Pallenis maritima* (L.) Greuter
16. *Parthenocissus quinquefolia* (L.) Planch
17. *Phyla canescens* (Kunth) Greene
18. *Solanum linnaeanum* Hepper & P.-M.L. Jaeger
19. *Tagetes minuta* L.
20. *Tropaeolum majus* L.
21. *Trigonella foenum-graecum* L.

S2.2. PERSISTING TAXA FROM CULTIVATION

1. *Bougainvillea* × *buttiana* Holttum & Standl
2. *Brachychiton acerifolius* (A. Cunn. ex G. Don) F. Muell.
3. *Firmiana simplex* W. Wight
4. *Pyrus malus* L. *subsp. mitis* (Wallr.) O. Bolòs & Vigo
5. *Salvia microphylla* Kunth
