# Supplementary material for: Montjuïc Hill (Barcelona): A Hotspot for Plant Invasions in a Mediterranean City
Source: Plants (Basel). 2023 Jul 21;12(14):2713. doi: 10.3390/plants12142713 (PMC10384852; doi:10.3390/plants12142713)
Supplement: Supplementary file 1 [file plants-12-02713-s001.zip › Text S3.pdf]

Text S3. Novelties for the catalogue of alien plant taxa of Montjuïc hill thanks to our fieldwork.

S3.1. NEW RECORDS FOR EUROPE OR THE IBERIAN PENINSULA AND BALEARIC ISLANDS

Bosea amherstiana Hook. f.

A mature subspontaneous bush was observed in grassy wasteland on Montjuïc (BC-956022, S. Pyke et al., 20/6/2016). It is a plant seldom cultivated in the Iberian Peninsula (Sánchez de Lorenzo, 2002) and is not known in a wild state in the rest of Europe (Randall, 2017).

Digitaria radicosa (J. Presl) Miq.

A taxon native to tropical Asia, appearing in areas of public gardens and, without doubt, introduced accidentally. The herborised plants from Montjuïc (BC-878038, 25/12/2011; BC-928015, 8/10/2012; BC-940048, 25/9/2014, S. Pyke) represent the first records of this plant, when appearing as a casual, in the Iberian Peninsula and probably also mainland Europe (Randall, 2017). It has been cited from Sardinia (Verloove, 2008) and the Canary Islands (Verloove, 2017).

Rumex lunaria L.

A bush observed recently in a wild state on Montjuïc, in an overgrown area above Costa i Llobera gardens (BC-956039, S. Pyke et al., 18/5/2016). It is not known to grow wild in the rest of the Iberian Peninsula, where it is occasionally cultivated as, for example, in Murcia (López, 2001; Sánchez de Lorenzo, 2014). It is native to the Canary Islands, and resistant to drought conditions, such as those of gardens with a low level of maintenance. Naturalised in Italy (Galasso *et al*., 2018) but no other records from the Iberian Peninsula are known.

S3.2. NOVELTIES FOR CATALONIA [NEW TAXA FOR CATALONIA]

Echium candicans L. f.

Observed in 2010 on the edge of a pine forest in the Joan Brossa gardens (S. Pyke, *vidi vivam*) and in 2017 a small group of plants near the south entrance of the Mossèn Costa and Llobera gardens (Gómez-Bellver *et al*., *vidi vivam*). Recently found a naturalized group at the foot of the hill on the side of Miramar (J. López-Pujol, 5/10/2020, *vidi vivam*).

Furcraea selloana K. Koch

["*selloa*" incorrect epithet according to Art. 60.8 International Code of Nomenclature Turland *et al*., 2018)]

Montjuïc, Miramar sector, below the road bordering Costa i Llobera gardens (J. López-Pujol & N. Nualart, 3/9/2020, *vidi vivam*) on steeply sloping ground.

Pandorea jasminoides (Lindl.) K. Schum.

Some escaped plants in flower on the maritime slopes of Montjuïc (BC-969047 and BCN-143183, C. Gómez-Bellver et al., 6/7/2017).

***Ruscus hypophyllum* L.**

A group of well-developed individuals observed in a woodland clearing on Montjuïc (BC-990453 and BCN-142966, P. Farelo, J. López-Pujol & C. Gómez-Bellver, 3/7/2017). This find confirms the presence of the taxon as a subspontaneous plant in Catalonia, although there is a record from 2001, from Sant Joan del Pas (Tarragona), reported by Royo (2006 —database of his thesis; ANTHOS, http://www.anthos.es/, retrieved at 20/7/2020) as «cultivated and ruderalised» which, nevertheless, does not appear in his thesis and has not been published. The historic records from Catalonia, some *ut R. hypoglossum*, appear to refer to cultivated plants.

S3.3. TAXA OBSERVED FOR THE FIRST TIME IN CATALONIA AT MONTJUÏC AND WHICH HAVE ALREADY BEEN PUBLISHED (AND THAT ARE ALREADY ON THE CATALONIAN CHECKLIST OF AYMERICH & SÁEZ, 2019)

Aristolochia sempervirens L.
About a dozen plants located in an area occupying approximately 1 km[2] between 130 and 180 m above sea level (Pyke, 2013).

Enneapogon cenchroides (Licht. ex Roem. & Schult.) C. E. Hubb.
A single specimen in full bloom in the castle moat's perimetral way (Gómez-Bellver *et al.*, 2016). New for Europe.

Morus kagayamae Koidz.
Subspontaneous individual on the edge of a ruderalized stream, north of the cemetery (Gómez-Bellver *et al.*, 2019[b]).

Opuntia elatior Mill.
Montjuïc Mountain on the Miramar side, a big subspontaneous plant, ca. 5–6 m wide, growing on a rocky slope (Gómez-Bellver *et al.*, 2019[a]).

Paraserianthes lophantha (Willd.) I.C. Nielsen
Several adult individuals with recruitment, on the path below the cemetery (Gómez-Bellver *et al.*, 2019[b]).

Ptelea trifoliata L.
Young individuals at three points in a pine forest around Montjuïc Castle (Gómez-Bellver *et al.*, 2019[b]). First citation in the Iberian Peninsula.

Tara spinosa (Feuillée ex Molina) **Britton & Rose [≡ *Caesalpinia spinosa*** (Feuillée ex Molina) Kuntze])
Group of large shrubs in a ruderal clearing in the freight port area of Barcelona (Gómez-Bellver *et al.*, 2019[b]).

Vachellia caven (Molina) Seigler & Ebinger
Subspontaneous young individual on Avinguda dels Muntanyans, next to the Historic Botanical Garden (Gómez-Bellver **et al.**, 2019[b]).

S3.4. SPECIFIC CASES

Succowia balearica

REFERENCES

Aymerich, P. & Sáez, L. 2019. Checklist of the vascular alien flora of Catalonia (northeastern Iberian Peninsula, Spain). *Mediterranean Botany* 40(2): 215-242.

Galasso, G.; Conti, F.; Peruzzi, L.; Ardenghi, N.M.G.; Banfi, E.; Celesti-Grapow, L. *et al*. 2018. An updated checklist of the vascular flora alien to Italy. *Plant Biosystems* 37 p.

Gómez-Bellver, C.; Álvarez, H. & Sáez, L. 2016. New contributions to the knowledge of the alien flora of the Barcelona province (Catalonia, Spain). *Orsis* 30: 167-189.

Gómez-Bellver, C.; Álvarez, H.; Nualart, N.; Ibáñez, N.; Sáez, L. & López-Pujol, J. 2019[a]. New records of vascular plants alien in Catalonia (NE Iberian Peninsula). *Collect. Bot.* 38: e004.

Gómez-Bellver, C.; Nualart, N.; Ibáñez, N.; Burguera, C.; Álvarez, H. & López-Pujol, J. 2019[b]. Noves dades de la flora al·lòctona de Catalunya i de la Comunitat Valenciana. *Butll. Inst. Cat. Hist. Nat*. 83: 23-40.

López, G. 2001. *Los árboles y arbustos de la Península Ibérica e Islas Baleares: especies silvestres y las principales cultivadas*. Mundi-Prensa Libros. 1727 p

Pyke, S. 2013. Notes on xenophytes detected in Catalonia, Spain. *Collect. Bot*. 32: 83-86.

Randall, R.P. 2017. *A Global Compendium of Weeds.* Third Edition. Perth, Western Australia. R.P. Randall. 2654 p.

Royo, F. 2006. Flora i vegetació de les planes i serres litorals compreses entre el riu Ebro i la serra d'Irta. Tesi Doctoral, Universitat de Barcelona, Barcelona.

Sánchez de Lorenzo, J.M. (cord.) 2002. *Flora Ornamental Española: las plantas cultivadas en la España peninsular e insular, II.* Cactaceae-Cucurbitaceae. Junta de Andalucía - Mundi Prensa. 667 p.

Sánchez de Lorenzo, J.M. 2014. *Rumex lunaria* L. (Polygonaceae), un arbusto de jardín muy poco conocido en Murcia. 3 p. <https://www.arbolesornamentales.es/Rumex%20lunaria%20en%20Murcia.pdf>

Turland, N. J., Wiersema, J. H., Barrie, F. R., Greuter, W., Hawksworth, D. L., Herendeen, P. S., Knapp, S., Kusber, W.-H., Li, D.-Z., Marhold, K., May, T. W., McNeill, J., Monro, A. M., Prado, J., Price, M. J. & Smith, G. F. (eds.) 2018: International Code of Nomenclature for algae, fungi, and plants (Shenzhen Code) adopted by the Nineteenth International Botanical Congress Shenzhen, China, July 2017. *Regnum Vegetabile* 159. Glashütten: Koeltz Botanical Books. DOI https://doi.org/10.12705/Code.2018

Verloove, F., 2008. Studies within the genus Digitaria Haller (Poaceae, Panicoideae) in southwestern Europe. *Candollea* 63: 227-233.

Verloove, F. 2017. New xenophytes from the Canary Islands (Gran Canaria and Tenerife; Spain). *Acta Bot. Croat*. 76(2): 120-131.
