# Supplementary material for: Montjuïc Hill (Barcelona): A Hotspot for Plant Invasions in a Mediterranean City
Source: Plants (Basel). 2023 Jul 21;12(14):2713. doi: 10.3390/plants12142713 (PMC10384852; doi:10.3390/plants12142713)
Supplement: Supplementary file 1 [file plants-12-02713-s001.zip › Text S4.pdf]

Text S4. Common taxa in Montjuïc hill that are rare in Catalonia.

1. *Aloe ferox* Mill.
2. *Aristolochia sempervirens* L.
3. *Brachychiton populneus* (Schott & Endl.) R. Br.
4. *Drosanthemum floribundum* (Haw.) Schwantes
5. *Lonicera fragrantissima* Lindl. & Paxton
6. *Morus kagayamae* Koidz.
7. *Opuntia monacantha* Haw.
8. *Oxalis bowiei* Aiton ex G. Don
9. *Paraserianthes lophantha* (Willd.) I.C. Nielsen
10. *Phytolacca dioica* L.
11. *Schinus molle* L.
12. *Senecio deltoideus* Less.
13. *Sinapis alba* L. subsp. *alba*
14. *Wigandia urens* (Ruiz & Pav.) Kunth
