# Supplementary material for: Montjuïc Hill (Barcelona): A Hotspot for Plant Invasions in a Mediterranean City
Source: Plants (Basel). 2023 Jul 21;12(14):2713. doi: 10.3390/plants12142713 (PMC10384852; doi:10.3390/plants12142713)
Supplement: Supplementary file 1 [file plants-12-02713-s001.zip › Text S5.pdf]

Text S5. Rare taxa in Montjuïc hill that are common in Catalonia.

1. *Bidens aurea* (Aiton) Sherff
2. *Brassica oleracea* L.
3. *Calendula officinalis* L.
4. *Celtis australis* L.
5. *Cuscuta campestris* Yunck.
6. *Echinochloa colona* (L.) Link
7. *Eragrostis curvula* (Schrad.) Nees
8. *Galinsoga parviflora* Cav.
9. *Helianthus annuus* L.
10. *Iris* × *germanica* L.
11. *Lathyrus tingitanus* L.
12. *Lunaria annua* L. subsp. *annua*
13. *Malus domestica* (Borkh.) Borkh.
14. *Melissa officinalis* L.
15. *Nassella neesiana* (Trin. & Rupr.) Barkworth
16. *Nothoscordum* × *borbonicum* Kunth
17. *Oenothera biennis* L.
18. *Oenothera rosea* Aiton
19. *Onobrychis viciifolia* Scop.
20. *Oxalis vallicola* (Rose) R. Knuth
21. *Parthenocissus inserta* (A. Kerner) Fritsch
22. *Paspalum vaginatum* Sw.
23. *Petasites pyrenaicus* (L.) G. López
24. *Phalaris canariensis* L.
25. *Pinus nigra* J.F. Arnold subsp. *nigra*
26. *Solanum lycopersicum* L.
27. *Vitis rupestris* Scheele
28. *Yucca gloriosa* L.
